# Lymphoma-Associated Biomarkers Are Increased in Current Smokers in Twin Pairs Discordant for Smoking

**DOI:** 10.3390/cancers13215395

**Published:** 2021-10-27

**Authors:** Jun Wang, David V. Conti, Marta Epeldegui, Miina Ollikainen, Rachel F. Tyndale, Amie Eunah Hwang, Larry Magpantay, Thomas McCulloch Mack, Otoniel Martinez-Maza, Jaakko Kaprio, Wendy Cozen

**Affiliations:** 1 Department of Preventive Medicine, Keck School of Medicine, University of Southern California, Los Angeles, CA 90089, USA; jun.wang@med.usc.edu (J.W.); dconti@usc.edu (D.V.C.); amiehwan@usc.edu (A.E.H.); tmack@med.usc.edu (T.M.M.); 2 Center for Genetic Epidemiology, Keck School of Medicine, University of Southern California, Los Angeles, CA 90089, USA; 3 Department of Obstetrics and Gynecology, AIDS Institute, David Geffen School of Medicine, University of California Los Angeles, Los Angeles, CA 90095, USA; MEpeldegui@mednet.ucla.edu (M.E.); LMagpantay@mednet.ucla.edu (L.M.); OMartinez@mednet.ucla.edu (O.M.-M.); 4 Department of Public Health, Faculty of Medicine, University of Helsinki, 00014 Helsinki, Finland; miina.ollikainen@helsinki.fi (M.O.); jaakko.kaprio@helsinki.fi (J.K.); 5 Centre for Addiction and Mental Health, Departments of Pharmacology & Toxicology, and Psychiatry, University of Toronto, and Campbell Family Mental Health Research Institute, Toronto, ON M5S 1A8, Canada; r.tyndale@utoronto.ca; 6 Department of Pathology, Keck School of Medicine, University of Southern California, Los Angeles, CA 90089, USA; 7 Department of Epidemiology, Fielding School of Public Health, University of California Los Angeles, Los Angeles, CA 90095, USA; 8 Microbiology and Immunology, David Geffen School of Medicine, University of California Los Angeles, Los Angeles, CA 90095, USA; 9 Institute for Molecular Medicine, University of Helsinki, 00014 Helsinki, Finland; 10 Department of Medicine, Division of Hematology-Oncology, School of Medicine, University of California Irvine, Orange, CA 92868, USA; 11 Department of Pathology, School of Medicine, University of California Irvine, Orange, CA 92868, USA; 12 Department of Epidemiology, School of Population Health, Susan and Henry Samueli College of Health Sciences, University of California Irvine, Irvine, CA 92697, USA

**Keywords:** TARC/CCL17, BAFF, MCP1, spg130, smoking, Hodgkin lymphoma, follicular lymphoma, twins, chemokines, cytokines, biomarkers

## Abstract

**Simple Summary:**

Smoking is associated with a moderate increased risk of Hodgkin and follicular lymphoma. To help understand why, we examined lymphoma-related biomarker levels among 134 smoking and non-smoking twins (67 pairs) ascertained from the Finnish Twin Cohort. We validated self-reported smoking history by measuring serum cotinine, a metabolite of nicotine, from previously collected frozen serum samples. In total, 27 immune biomarkers were assayed using the Luminex Multiplex platform (R & D Systems). We found that four immune response biomarkers were higher and one was lower among smoking compared to non-smoking twins. The strongest association was observed for CCL17/TARC, a biomarker elevated in Hodgkin lymphoma patients. Immune biomarker levels were similar in former smokers and non-smokers. Current smoking may increase levels of immune proteins that could partially explain the association between smoking and risk of certain lymphomas.

**Abstract:**

Smoking is associated with a moderate increased risk of Hodgkin and follicular lymphoma. To understand why, we examined lymphoma-related biomarker levels among 134 smoking and non-smoking twins (67 pairs) ascertained from the Finnish Twin Cohort. Previously collected frozen serum samples were tested for cotinine to validate self-reported smoking history. In total, 27 immune biomarkers were assayed using the Luminex Multiplex platform (R & D Systems). Current and non-current smokers were defined by a serum cotinine concentration of >3.08 ng/mL and ≤3.08 ng/mL, respectively. Associations between biomarkers and smoking were assessed using linear mixed models to estimate beta coefficients and standard errors, adjusting for age, sex and twin pair as a random effect. There were 55 never smokers, 43 current smokers and 36 former smokers. CCL17/TARC, sgp130, haptoglobin, B-cell activating factor (BAFF) and monocyte chemoattractant protein-1 (MCP1) were significantly (*p* < 0.05) associated with current smoking and correlated with increasing cotinine concentrations (*P*_trend_ < 0.05). The strongest association was observed for CCL17/TARC (*P*_trend_ = 0.0001). Immune biomarker levels were similar in former and never smokers. Current smoking is associated with increased levels of lymphoma-associated biomarkers, suggesting a possible mechanism for the link between smoking and risk of these two B-cell lymphomas.

## 1. Introduction

Smoking is an established risk factor for multiple epithelial cancers [1]. Accumulating evidence suggests that it may also increase the risk of some hematological cancers. Cigarette smoking is associated with a modestly increased overall risk of Hodgkin lymphoma (HL), but approximately 60–80% increased risk for certain HL subtypes, including mixed cellularity HL and Epstein–Barr virus (EBV)-positive HL [2,3]. It is also a risk factor for follicular lymphoma, one of the common subtypes of non-Hodgkin Lymphoma (NHL) [4]. However, the exact mechanism underlying the association between cigarette smoking and increased lymphoma risk remains largely unexplained. Cigarette smoking can alter immune responses by producing Th2 cytokines, including interleukin [IL]4 (IL4) and IL13 [5], which may protect B-cell tumors against apoptosis [6]. IL13 levels are also higher among infants exposed to prenatal maternal smoking compared to those without smoking exposure [7]. In a convenience sample, Byron and colleagues showed that phytohemagglutinin (PHA) induced IL4 production in cultured peripheral blood mononuclear cells (PBMC) of smokers was significantly higher compared to that in non-smokers [8]. We used the same laboratory method applied to a set of monozygotic (MZ) twin pairs with varying levels of smoking, naturally matched on genetics, early life and parental smoke exposure, and found that serum IL13 supernatant levels from similarly PHA-stimulated PBMCs were significantly higher among smoking compared to non-smoking twins [9]. PHA-stimulation was used to obtain measurable levels of these cytokines, which are often undetectable and unstable in serum. The method artificially increased levels of these Th2 cytokines and thus physiological responses were not evaluated. A recent tobacco industry-funded study examined leukocyte subset counts by flow cytometry and serum levels of inflammatory cytokines among Italian smoking-discordant twin pairs and found differences in leukocyte counts but not serum cytokine levels [10]. They did not examine soluble immune biomarkers related to HL or FL and did not account for smoking duration or current vs. former smoking.

Altered immunity plays a critical role in both HL and NHL risk and pathogenesis [11,12,13,14], in which both immune deficiency and immune activation are associated with increased risk. Pre-diagnostic serum levels of markers of B-cell activation (i.e., sCD23, sCD27, sCD30 and sCXCL13), even as early as 9 years before diagnosis, were significantly associated with increased NHL risk in the Women’s Health Initiative Cohort study [15]. CCL17/TARC, CD30 and IL6 are among the immune-mediating molecules associated with HL prognosis and pathogenesis [16,17,18].

In addition, microbial translocation of bacterial molecules (e.g., endotoxin from Gram-negative bacterial cell walls) from the gut to bloodstream through a leaky intestinal epithelium results in excessive macrophage stimulation, which, in turn, induces B-cell stimulation, with low levels of endotoxin promoting B-cell induced CD4+ proliferation and high levels promoting CD8+ proliferation [19]. Endotoxin induces B-cell activating factor (BAFF), which is associated with lymphomagenesis [20]. We measured serum markers of microbial translocation (haptoglobin, lipopolysaccharide-binding protein (LPB), fatty acid binding protein 2 (FABP2) and soluble CD14) and macrophage activation (BAFF, CCL17/TARC, monocyte chemoattractant protein-1 (MCP1), and the soluble form of the 130 kDa IL6 receptor (s13gp0)) in a cohort of HIV-positive patients and found that higher levels at baseline predicted the development of NHL [21]. Because smoking disrupts the alveolar–blood barrier through physical assaults of heat and through a variety of chemical exposures in tobacco, microbial translocation occurs at this site as well [22], and thus could conceivably be a mechanism that increases risk of NHL or HL by allowing endotoxins to enter the bloodstream to promote immune dysregulation.

To better understand the association between smoking and lymphoma risk, we sought to characterize immune markers related to lymphoma in a sample of MZ from the Finnish Twin Cohort [23] study with varying levels of cigarette smoking. Twins as participants bring advantages to the study of environmental and lifestyle exposures because they are matched in terms of genetics and early life environment and therefore mitigate potential confounding by these factors when comparing unrelated cases and controls.

## 2. Materials and Methods

### 2.1. Study Population

In total, 67 MZ twin pairs (134 individual twins) with no major health conditions such as cardiovascular disease or cancer were identified from three longitudinal population-based Finnish twin cohorts designed to investigate genetic and environment determinants of common diseases and related behavioral risk factors. The older Finnish Twin Cohort consists of adult twins born from 1938 to 1957, the FinnTwin12 [24] comprises twins born from 1983 to 1987 and the FinnTwin16, from 1975 to 1979. The details of these twin cohorts have been described previously [23,24,25]. All participants provided written informed consent. The study was approved by the Ethics Committee of the Helsinki University Central Hospital and the University of Southern California Health Sciences Campus Institutional Review Board.

Participants’ demographics and self-reported lifetime smoking information, including age at initiation and cessation and quantity, were ascertained through structured questionnaires and interviews. Twins provided a baseline fasting blood sample, which was collected, processed and stored as serum at the time of enrollment. Monozygosity was confirmed using genetic markers.

### 2.2. Cotinine and Immune-Related Biomarker Measurement

Cotinine was measured from frozen serum samples using liquid chromatography/tandem mass spectrometry as previously described [26]. Thirty-nine immune related biomarkers capturing inflammation, B-cell activation and microbial translocation were measured in this study. Twelve biomarkers (IL1 beta [IL1b], IL2, IL5, IL13, IL17A, IL17F, IL22, IL23, interferon gamma [IFNg], IL4, IL10, IL12) were excluded due to undetectable results in at least 12% of the subjects. Twenty-seven biomarkers were included in the final analysis, representing inflammation-associated molecules (IL6, soluble IL6 receptor alpha [sIL6RA], IL8, IL18, IL1 receptor antagonist [IL1RA], interferon gamma-induced protein 10 [IP10/CXCL10], monocyte chemoattractant protein-1 [MCP1], soluble 130 kDa IL6 receptor glycoprotein [sgp130], tumor necrosis factor alpha [TNFA], soluble tumor necrosis factor alpha receptor type II [sTNFRII]), Th2-associated immune system molecules (B-cell activating factor [BAFF], C-C motif chemokine ligand 17 [CCL17]/thymus and activation regulated chemokine [TARC], C-C motif chemokine 22 [CCL22], C-C-C-C motif ligand 24 [CCL24], C-X-C motif chemokine ligand 13 [CXCL13], and soluble IL2R alpha [sIL2RA]) and microbial translocation (soluble CD14 [sCD14], sCD163, EndoCab IgM, fatty acid-binding protein 2 [FABP2], haptoglobin [HP], lipopolysaccharide binding protein [LBP]), and other functions (sCD30, FABP4, fibroblast growth factor 21 [FGF21], intercellular adhesion molecule 1 [ICAM1], IL15). All biomarkers, except HP and EndoCab, were measured using the Luminex xMAP system, which allowed the multiplexed simultaneous quantification of up to 100 biomarkers per panel, as previously described [15,21,27]. This system uses spectrally addressed bead sets, each of which is conjugated with a different capture monoclonal antibody specific for a given target molecule. The bead mixtures were analyzed using a BioPlex 200 Luminex machine, to quantify the signal per bead address, and to provide levels of these analytes. Kits for the individual assays are from R & D Systems (Minneapolis, MN, USA). HP was measured with a standard ELISA assay from R & D Systems and EndoCab IgM was measured by ELISA from Hycult Biotech. Values below the detectable level, as provided by the assay instructions, were excluded in the analysis. Six biomarkers (CCL17/TARC, TNFA, FGF21, EndoCAB, HP and IL6) had undetectable levels in 0.7% to 11.9% of the specimens.

### 2.3. Statistical Analysis

The primary purpose of the analysis was to examine the effect of tobacco smoking on the levels of specific immune response proteins (biomarkers) related to lymphoma, using a set of matched twins to mitigate potential confounding for a more valid assessment of the effect. We assessed cigarette smoking exposure primarily using serum cotinine concentrations, with additional information on never and former smoking ascertained by self-report. Current smoking was defined as cotinine >3.08 ng/mL and non-current as cotinine ≤3.08 ng/mL based on previous studies [28]. Thirty-one twin pairs were discordant for current smoking and the remaining pairs were concordant for smoking status (30 pairs were concordant non-current smokers and 6 were concordant for current smoking, 4 with different amounts of smoking). We first assessed the association between current vs. non-current smoking status and each of the 27 biomarkers. We performed sensitivity analyses examining each of the 27 measurable biomarkers separately for never and former smokers compared to current smokers. We then retained only biomarkers that were significantly associated with current smoking status (*p* < 0.05) for further assessment. Classification of never and former smoking status was based on a self-report in the questionnaire and a serum cotinine ≤3.08 ng/mL (twins who reported that they were former smokers but had serum cotinine >3.08 ng/mL were re-classified to current smokers, assuming misreporting). We combined never and former smokers as a single reference group of “non-current smokers” for most analyses, unless otherwise specified, assuming that the effect of smoking on the immune system was not permanent and would occur in the setting of current, not past, exposure.

In subsequent analyses, we evaluated (1) whether smoking cessation and time since quitting impacted biomarker levels and (2) the dose–response relationship between serum cotinine and immune biomarker levels among current smokers. To assess a dose–response association between serum cotinine on biomarker levels, we further categorized current smokers (those with cotinine >3.08) into “lighter” vs. “heavier” cotinine based the median cotinine level (78.17 ng/mL) in our sample.

For the statistical analysis, we used linear mixed models, adjusting for twin pairs via an intercept, which provided both within and between twin pair information to estimate the beta coefficients and standard errors [29], equivalent to a paired *t*-test in linear regression [30]. By using MZ twins in this study, we have the added advantage of controlling for familial, in utero and other early life confounders not possible in a study of unrelated individuals. Age and sex were adjusted in the mixed models as fixed effects and a term for twin pair was included as a random effect, as previously described [9]. Biomarkers were log2 transformed due to the skewed distribution except BAFF, CCL22, sCD14, sgp130 and IL15, because they had a normal distribution. We investigated adjustment for body mass index (BMI) given the correlation between smoking and obesity in prior studies [31,32] but did not find that BMI was related to smoking in our study and thus did not adjust for it in the mixed models. Least-squares (LS) means or geometric means were compared between smoking levels and *p* values were adjusted using Tukey’s multiple comparison method.

## 3. Results

The mean and median age of the participating twins were 37.3 years (SD: 18.9) and 24.8 years (range: 21.0–68.9), respectively (Table 1). More than half of the twins were females (56.7%) and most were non-current smokers (67.9%). Approximately 41% and 27% of twins were never and former smokers, respectively. Among former smokers, 44% of them had quit smoking more than 5 years ago. By twin pair, 36 (35.6%) were non-smokers/former smokers, 29 (28.7%) were non-smokers/current smokers, 16 (15.8%) were current smokers/former smokers, 18 (17.8%) were concordant for current smoking and 2 (2.0%) were concordant for former smoking.

**Figure 1:**
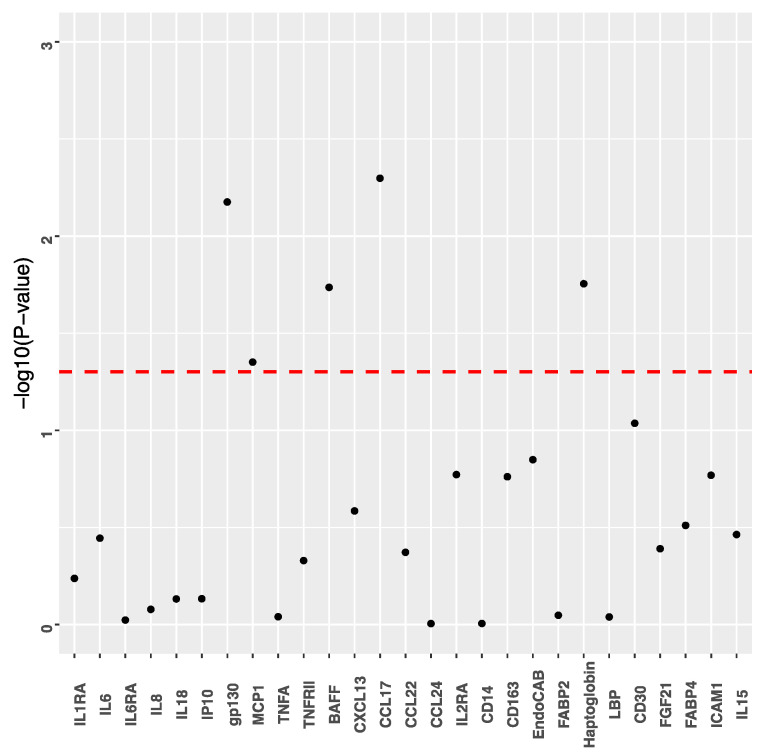
Associations between serum cotinine and each individual immune-related biomarker (*N* = 27). Cotinine levels (ng/mL) are defined as noncurrent (≤3.08) and current (>3.08). Dots represent *p* values from mixed models assessing cotinine (> vs. ≤3.08) and individual biomarker separately. Red dashed line represents *p* = 0.05.

Out of the 27 immune-related biomarkers, 5 were significantly (*p* < 0.05) associated with current compared to non-current smoking: CCL17/TARC, sgp130, HP, BAFF and MCP1 (Figure 1 and Table S1).

The strongest association was seen for CCL17/TARC (*P*_trend_ = 0.0001), assessed in 85/91 participants. Twins who were current heavier smokers (defined by serum cotinine concentration above the median in this sample) had a mean CCL17/TARC level that was 52.4% higher than current lighter smoking twins (*P*_adjusted_ = 0.006), and 62.9% higher than non-current smoking twins (*P*_adjusted_ = 0.0001) (Table 2). Heavier smokers also had statistically significantly higher mean levels of BAFF (8.9%, *P*_adjusted_ = 0.02), haptoglobin (29.0%, *P*_adjusted_ = 0.04) and MCP1 (22.0%, *P*_adjusted_ = 0.08.) compared to current non-smokers (Table 2). In contrast, the mean sgp130 level for current heavier smokers was 6.3% lower than the mean level in non-current smokers (*P*_adjusted_ = 0.03) (Table 2). When immune biomarker levels were compared in heavier smokers to lighter smokers, and lighter smokers to non-current smokers, the directions of the associations were the same, but differences did not reach statistical significance (Table 2).

**Figure 2:**
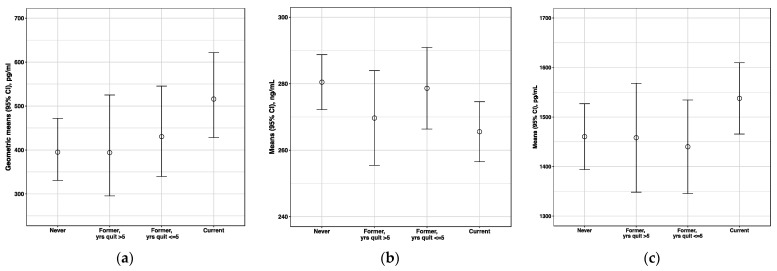
Associations between smoking status and biomarker levels: (**a**) CCL17/TARC (*P*_trend_ = 0.01); (**b**) sgp130 (*P*_trend_ = 0.01); (**c**) BAFF (*P*_trend_ = 0.08). Smoking status and years since quit were self-reported; however, twin pairs with discordance in self-reports and serum cotinine levels were excluded from analysis. Points represent means (BAFF and sgp130) or geometric means (CCL17/TARC) of biomarker levels and solid lines represent 95% confidence interval (CI).

We observed a significant dose–response relationship between smoking levels (current heavier, current lighter and non-current smoking) and the five biomarker levels (*P*_trend_ < 0.05; Table S2). Circulating levels of the five immune biomarkers among self-reported former smokers were not significantly different from those in never smokers (Table S3a); however, they were significantly different compared to those in current smokers (Table S3b). When former smokers were examined in further detail, a trend was observed between years since quitting and levels of CCL17/TARC (positive) and sgp130 (inverse) (*P*_trend_ < 0.05; Table S4). There was a similar but borderline significant trend for BAFF (*P*_trend_ = 0.08; Table S4). Former smokers who quit more than five years ago had geometric mean CCL17/TARC levels similar to those of never smokers, while those who quit more recently had levels that were intermediate between never smokers and current smokers (*P*_trend_ = 0.01) (Figure 2). Former smokers generally had sgp130 and BAFF levels that were intermediate between never and current smokers; however, the differences by time since quitting were not as evident.

There was a significant trend for immune biomarker levels according to the duration of smoking (≤5 years, >5 years), with the exception of MCP1 (Table S5). Relative to the reference group of non-current smokers, beta values were increased in longer duration compared to shorter duration smokers. All five immune biomarkers were significantly associated with self-reported smoking of ≥10 cigarettes per day but not with <10 cigarettes per day (Table S6), consistent with observations when serum cotinine concentration above and below the median concentration was used to define heavier and lighter smoking. There was a moderate positive correlation between HP and BAFF (Pearson *r* = 0.26, *p* < 0.05) and CCL17/TARC (Pearson *r* = 0.2, *p* < 0.05) (Figure S1), but not between the other biomarkers.

We examined the intra-pair correlation coefficient (ICC) for the 27 biomarkers among the 67 MZ twin pairs and observed that approximately 60% of the biomarkers demonstrated ICC >0.5 (Table S7). Of the five immune biomarkers identified in this study, CCL17/TARC, BAFF and sGP130 all showed an ICC >0.5 while haptoglobin and MCP1 demonstrated a lower ICC (ranging from 0.28–0.44). Specifically, the ICC for CCL17/TARC was 0.66 (95%CI: 0.50–0.78).

## 4. Discussion

Immune responses, nicotine metabolism pathways and smoking behaviors are at least partially heritable [33,34,35]. We took advantage of natural genetic matching in addition to age-, sex-, early life in twins to conduct a unique study to examine the effect of an exposure (smoking) on immune biomarkers related to lymphoma. To enhance the validity of the matched comparison and limit confounding by genetics, we limited the study to MZ twins only. We found that current smoking status, defined by serum cotinine levels, was significantly associated with serum levels of markers related to inflammation and macrophage activation (MCP1 and sgp130), Th2 immune response (CCL17/TARC and BAFF) and microbial translocation (haptoglobin) pathways, markers that are associated with both HL and NHL [15,21,36]. For several of these markers, including CCL17/TARC, levels among former smokers who stopped smoking over 5 years ago were similar to those in never-smokers, suggesting that smoking cessation may lead to a reversal of smoking-induced biomarker alterations. We did not observe differences in innate inflammatory immune biomarkers including IL8, TNFα and sTNFRII, consistent with a previous study that examined these factors in 22 pairs of smoking-discordant twins [10] (other soluble markers associated with HL, FL and microbial translocation examined in our study were not examined in this previous twin study).

Although our numbers were small, the results were statistically significant and there was strong biological plausibility. The strongest association we observed was between current smoking and increased CCL17/TARC levels, consistent with results from a prior nested case–control study of unrelated individuals [37]. In that study, smokers had a four-fold increased risk of high serum CCL17/TARC levels compared to non-smokers, but no dose–response association with cigarettes per day was observed [37]. In an experimental animal model of allergic asthma, exposure to cigarette smoke induced CCL17/TARC production and secretion in macrophages from bronchial lavage, evidence that smoke exposure appears to be causing the increase in CCL17/TARC [38]. CCL17/TARC is a confirmed biomarker for HL, is highly expressed by the neoplastic Hodgkin–Reed–Sternberg (HRS) cells with elevated levels found in the majority of HL patients [39,40]. Smoking is associated with an increased risk of overall HL, and even more strongly associated with mixed cellularity and EBV+ subtypes [2,3]. Our results, along with the previous study of unrelated individuals [37], suggest that the higher levels of CCL17/TARC in smokers may contribute to the association between HL and smoking.

Three of the other four markers (MCP1, BAFF and haptoglobin) were positively associated with smoking and one (sgp130) was inversely associated. MCP1, HP and BAFF are linked to smoking in specific disease contexts and different tissues (e.g., bronchial secretions [41,42,43], and sgp130 has been linked indirectly to smoking via the STAT3 transcription factor pathway and modulation of tobacco carcinogen induced lung cancer [44]. There is experimental evidence that smoking can induce MCP1, CCL17/TARC, BAFF production, primarily in macrophages [38,43,45]. Thus, there is previous evidence suggesting that smoking can activate these cytokines/chemokines in macrophages, which could then promote B-cell activation, leading to lymphomagenesis.

Haptogloblin is a molecule produced in lung tissue (among others) that binds to hemoglobin and thus decreases oxygenation. Its target is CD163 on macrophages, where it induces phagocytosis to remove it from tissues to limit damage from free heme [46]. The association between elevated levels of haptogloblin and smoking suggests that perhaps that injury to the alveolar membranes results in increased secretion of this acute phase protein. Elevated levels of this marker predict AIDS-associated non-Hodgkin lymphoma in the setting of microbial translocation, where Gram-negative bacteria leak into the bloodstream from damaged intestinal epithelium [47]. Other immune markers associated with microbial translation were not associated with smoking in this study, providing little support for this pathway.

Levels of CCL17/TARC, sgp130 and BAFF in those who quit smoking over five years ago were similar to those who never smoked, suggesting the effect of smoking on these biomarkers is not permanent. This is consistent with the epidemiological observation that current, but not former, smokers had an increased risk of HL [2]. In an earlier study, the same group found that risk of HL decreased significantly as time since quitting increased [48]. For follicular lymphoma, the 10% increased risk associated with smoking appears to be maintained for the first 10 years after quitting, but then risk decreases slowly after that [4]. Larger longitudinal studies that assess the biomarker changes corresponding to smoking cessation are warranted to better characterize the trajectories of smoking cessation on biomarker levels.

Our study has several strengths. A major advantage is the use of genetically identical twin pairs as the study population (i.e., within-pair analyses, see Methods). Circulating immune-related markers can be influenced by genetic variation [49]; thus, adjustment for twin pair can control for the genetic factors in the assessment of the smoking and biomarker association. Of the 27 biomarkers examined in this study, 60% demonstrated ICC >0.5 in all the MZ twin pairs, suggesting at least moderate genetic influence on the biomarker levels. CCL17/TARC, the strongest association identified in this study, showed an ICC 0.66 (95%CI: 0.50–0.78). Thus, using genetically identical twin pairs as study population made it possible to control for genetic influence on biomarker levels. Moreover, inherent matching on parental smoke exposure, age and sex in twins controls for additional cofounding. Another strength of our study is the use of serum cotinine concentration to quantify current exposure to tobacco [28], more objectively assessing current smoking compared to self-reported tobacco use (although some analyses showed similar results for cotinine and self-reported smoking amount). Finally, we conducted a novel comprehensive investigation of the effects of smoking on lymphoma-associated circulating immune-related markers.

Our study also has several limitations, especially small sample size for smoking concordant twin pairs, which precluded a more detailed assessment of the dose–response effect of cumulative smoking exposure (e.g., years of smoking) on circulating immune biomarker levels. Because the subjects were all Finnish twins of European origin, the lack of cultural, racial and ethnic diversity limits generalizability. Racial/ethnic differences in nicotine metabolism are well established and slow metabolizers are uncommon in populations of European origin [28]. Some systemic inflammatory markers, such as C-reactive protein, vary by racial and ethnic groups [50]. Nevertheless, our study provides new information that can be validated in other groups in the future.

## 5. Conclusions

In conclusion, our results suggest that current smoking may be associated with altered levels of immune proteins associated with B-cell and macrophage activation and immune cell trafficking that could explain the association between HL, and possibly FL, and smoking. The strongest association was between current smoking and higher circulating levels of TARC/CCL17, an established HL disease biomarker. Importantly, levels of the 5 significantly associated immune proteins were similar in never smokers and those who had long since quit smoking, suggesting a relatively short-term effect of tobacco on the immune system. We previously demonstrated an association between smoking and levels of Th2 proteins (IL-4, IL-5 and IL-13) secreted in supernatant from PHA-stimulated T-cells in smoking discordant twins [9], an artificial system. We also showed that current and former smokers had higher persistent antibody levels to *C. pneumoniae*-specific serum IgA compared to their non-smoking twins [51], but the immune response was in the context of a specific infection. To our knowledge, the present study is the first that demonstrates an elevation of immune proteins relevant to lymphomagenesis in serum from genetically matched twin smokers and non-smokers.

## Figures and Tables

**Table 1:** Characteristics of participating monozygotic (MZ) twins from the Finnish Twin Cohort Study.

Characteristics	Measure (SD or %)
	67 Twin Pairs
Mean Age (SD)	37.3 (18.9)
Female, N (%)	76 (56.7)
	134 Individual Twins
BMI kg/m^2^, mean (SD)	25.0 (4.7)
Serum cotinine ng/mL, N (%)	
≤3.08 (Non-current smoking)	91 (67.9)
>3.08 (Current smoking)	
Low	22 (15.7)
High	21 (16.4)
Smoking status ^1^, N (%)	
Never	55 (41.0)
Former	36 (26.9)
Current	43 (32.1)
Years since quit among former smokers, N (%)	
≤5	20 (55.6)
>5	16 (44.4)

^1^ Defined according to both serum cotinine and self-reported smoking status.

**Table 2:** Differences in immune biomarker levels by smoking amount defined by serum cotinine concentration.

Smoking Level Comparison ^1^	Difference in Least Squares Means ^2^	% Difference	*p*	*P*_adj_^3^
BAFF, pg/mL				
Current lighter smokers–Noncurrent	48.2	3.3	0.27	0.51
Current heavier smokers–Current lighter smokers	80.7	5.4	0.17	0.35
Current heavier smokers–Noncurrent	128.9	8.9	0.01	0.02
CCL17, pg/mL				
Current lighter smokers–Noncurrent	27.4	6.9	0.51	0.78
Current heavier smokers–Current lighter smokers	223.3	52.4	0.002	0.006
Current heavier smokers–Noncurrent	250.6	62.9	<0.0001	0.0001
Haptoglobin, ng/mL				
Current lighter smokers–Noncurrent	90.3	12.5	0.22	0.43
Current heavier smokers–Current lighter smokers	119.5	14.7	0.28	0.53
Current heavier smokers–Noncurrent	209.8	29.0	0.02	0.04
sgp130, ng/mL				
Current lighter smokers–Noncurrent	−10.0	−3.6	0.11	0.24
Current heavier smokers–Current lighter smokers	−7.5	−2.8	0.35	0.62
Current heavier smokers–Noncurrent	−17.5	−6.3	0.01	0.03
MCP1, pg/mL				
Current lighter smokers–Noncurrent	30.4	8.4	0.35	0.61
Current heavier smokers–Current lighter smokers	50.1	12.8	0.28	0.52
Current heavier smokers–Noncurrent	80.5	22.3	0.03	0.08

^1^ For BAFF and sgp130, differences in means were presented; for CCL17/TARC, Haptoglobin and MCP1, differences in geometric means were presented. Means or geometric means were obtained from mixed models adjusted for age and sex. ^2^ Serum cotinine levels (ng/mL): Non-current (cotinine ≤ 3.08), current lighter smokers (cotinine: 3.08–78.17), current heavier smokers (cotinine > 78.17). ^3^ *p*-values adjusted using Tukey’s method for multiple comparison adjustment.

## Data Availability

The twin dataset used in the current study will be located in the Biobank of the National Institute for Health and Welfare, Finland. All the biobanked data are publicly available for use by qualified researchers following a standardized application procedure (https://thl.fi/en/web/thl-biobank/for-researchers accessed on 25 November 2021).

## Supplementary Materials

The following are available online at https://www.mdpi.com/article/10.3390/cancers13215395/s1, Table S1: The association between serum cotinine defined smoking status (non-current vs. current) and biomarker levels, Table S2. The association of serum cotinine and immune biomarkers levels, Table S3: The association between smoking status (never/former/current) and serum immune biomarker levels. Never smokers (a) or former smokers (b) were used as reference group, Table S4: The association of self-reported smoking status and serum immune biomarkers levels, Table S5: The association of years of smoking and serum immune biomarkers levels, Table S6: The association of cigarettes per day (CPD) and serum immune biomarkers levels, Table S7: Biomarker intra-pair correlation coefficient (ICC) in the 67 MZ twin pairs, Figure S1: Pearson correlation of serum biomarkers.
