# Supplementary material for: Lymphoma-Associated Biomarkers Are Increased in Current Smokers in Twin Pairs Discordant for Smoking
Source: Cancers (Basel). 2021 Oct 27;13(21):5395. doi: 10.3390/cancers13215395 (PMC8582438; doi:10.3390/cancers13215395)
Supplement: Supplementary file 1 [file cancers-13-05395-s001.zip › cancers-1405106-suppl-new.pdf]

Supplementary Materials: Lymphoma-Associated Biomarkers are Increased in Current Smokers in Twin Pairs Discordant for Smoking

Jun Wang, David V. Conti, Marta Epeldegui, Miina Ollikainen , Rachel F. Tyndale, Amie Eunah Hwang, Larry Magpantay, Thomas McCulloch Mack, Otoniel Martinez-Maza, Jaakko Kaprio and Wendy Coze

Table S1. The association [1] between serum cotinine defined smoking status (non-current vs. current) [2] and biomarker levels [3].

Variable	Non-Current Smoking (Ref)	Current Smoking			
	N	*N*	Beta	SE	*P*
CCL17	85	43	0.35	0.12	0.005
sgp130	91	43	−13.33	4.92	0.007
Haptoglobin	85	41	0.26	0.11	0.02
Baff	91	43	83.20	35.28	0.02
MCP1	91	43	0.20	0.10	0.04
CD30	91	43	−0.12	0.07	0.09
Endocab	85	41	−0.10	0.07	0.14
IL2RA	91	43	0.09	0.06	0.17
ICAM1	91	43	0.14	0.10	0.17
CD163	91	43	−0.15	0.11	0.17
CXCL13	91	43	0.14	0.13	0.26
FABP4	91	43	−0.23	0.22	0.31
IL15	91	43	0.07	0.08	0.34
IL6	73	37	0.26	0.28	0.36
FGF21	90	42	−0.23	0.28	0.41
CCL22	91	43	39.59	49.57	0.42
TNFRII	91	43	−0.03	0.04	0.47
IL1RA	91	43	0.06	0.10	0.58
IP10	91	43	0.03	0.08	0.74
IL18	91	43	0.04	0.11	0.74
IL8	91	43	0.02	0.11	0.83
FABP2	91	43	−0.02	0.12	0.90
TNFA	84	40	−0.02	0.18	0.91
LBP	91	43	−0.01	0.08	0.91
IL6RA	91	43	−0.002	0.03	0.95
CD14	91	43	−0.41	25.33	0.99
CCL24	91	43	−0.001	0.07	0.99

[1] Adjusted for age and sex in the mixed models. Except BAFF, CCL22, CD14, sgp130 and IL15, all the biomarkers were run as log2 transformed in the mixed models. [2] Non-current smoking: serum cotinine ≤3.08 ng/mL and current smoking: serum cotinine >3.08 ng/ml. [3] The unit of all biomarkers is pg/ml, except CD14 and gp130 as ng/ml.

Table S2. The association [1] of serum cotinine [2] and immune biomarkers levels [3].

Variable	Non-Current (Ref)	Current, Low Cotinine				Current, High Cotinine				P_{trend}
	N	***N***	**Beta**	**SE**	***P***	***N***	**Beta**	**SE**	***P***	
CCL17	85	22	0.10	0.14	0.50	21	0.70	0.16	8.76E-06	0.0001
sGP130	91	22	−10.00	5.98	0.09	21	−17.52	6.58	0.01	0.004
Baff	91	22	48.22	42.74	0.26	21	128.87	47.39	0.01	0.01
Haptoglobin	85	21	0.17	0.13	0.20	20	0.37	0.15	0.01	0.01
MCP1	91	22	0.12	0.12	0.33	21	0.29	0.13	0.02	0.02

[1] Adjusted for age and sex in the mixed models. Except BAFF and gp130, all the biomarkers were run as log2 transformed in the mixed models. [2] Serum cotinine levels (ng/mL): Non-current (cotinine ≤ 3.08), current low (cotinine: 3.08–78.17), current high (cotinine >78.17). [3] The unit of all biomarkers is pg/ml, except gp130 as ng/ml.

Table S3. a and b. The association [1] between smoking status (never/former/current) and serum immune biomarker levels [2]. Never smokers (a) or former smokers (b) were used as reference group.

a.

Variable	Never(Ref)	Former				Current			
	N	*N*	Beta	SE	*P*	*N*	Beta	SE	*P*
sGP130	55	36	−5.90	5.14	0.25	43	−15.59	5.26	0.003
CCL17	52	33	0.07	0.13	0.60	43	0.38	0.13	0.01
BAFF	55	36	−12.13	36.79	0.74	43	78.66	37.87	0.04
Haptoglobin	52	33	−0.07	0.12	0.56	41	0.23	0.12	0.05
MCP1	55	36	−0.07	0.11	0.48	43	0.17	0.11	0.12

b.

Variable	Former (ref)	Never				Current			
	N	*N*	Beta	SE	*P*	*N*	Beta	SE	*P*
Baff	36	55	12.13	36.79	0.74	43	90.79	41.85	0.03
MCP1	36	55	0.07	0.11	0.48	43	0.24	0.12	0.03
sGP130	36	55	5.90	5.14	0.25	43	−9.70	5.78	0.09
CCL17	33	52	−0.07	0.13	0.60	43	0.31	0.15	0.04
Haptoglobin	33	52	0.07	0.12	0.56	41	0.30	0.13	0.02

[1] Adjusted for age and sex in the mixed models. Except BAFF and gp130, all the biomarkers were run as log2 transformed in the mixed models. [2] The unit of all biomarkers is pg/ml, except gp130 as ng/ml.

Table S4. The association [1] of self-reported smoking status and serum immune biomarkers levels [2].

Variable	Never (Ref)	Former, Years Since Quit >5				Former, Years Since Quit <=5				Current				P_{trend}
	N	*N*	Beta	SE	*P*	*N*	Beta	SE	*P*	*N*	Beta	SE	*P*	
Baff	55	16	−2.20	52.46	0.97	20	−20.45	48.08	0.67	43	77.22	38.23	0.04	0.08
sgp130	55	16	−10.80	7.22	0.13	20	−1.84	6.66	0.78	43	−14.91	5.28	0.005	0.01
MCP1	55	16	−0.18	0.15	0.22	20	0.02	0.14	0.89	43	0.18	0.11	0.09	0.15
CCL17	52	13	−0.004	0.20	0.98	20	0.12	0.17	0.47	43	0.38	0.14	0.005	0.01
Haptoglobin	52	13	0.07	0.17	0.68	20	−0.17	0.15	0.25	41	0.21	0.12	0.07	0.15

[1] Adjusted for age and sex in the mixed models. All biomarkers except BAFF and sgp130 were run as log2 transformed in the mixed models. [2] The unit of all biomarkers is pg/ml, except sgp130 as ng/ml.

Table S5. The association [1] of years of smoking and serum immune biomarkers levels [2].

Variable	Non-Current (Ref)	Current, Years ≤5				Current, Years > 5				
	N	*N*	Beta	SE	*P*	*N*	Beta	SE	*P*	P_{trend}
CCL17	81	17	0.23	0.16	0.15	14	0.67	0.18	2.12E-04	0.0002
Baff	87	17	19.69	46.27	0.67	14	209.04	52.98	7.95E-05	0.0005
Haptoglobin	81	17	0.27	0.13	0.04	14	0.42	0.15	0.004	0.001
sgp130	87	17	−10.44	6.50	0.11	14	−15.93	7.34	0.03	0.01
MCP1	87	17	0.09	0.14	0.53	14	0.23	0.15	0.14	0.12

[1] Adjusted for age and sex in the mixed models. All biomarkers except BAFF and sGP130, were log2 transformed in the mixed models. [2] The unit of all biomarkers is pg/mL, except sgp130 as ng/mL.

Table S6. The association [1] of cigarettes per day (CPD) and serum immune biomarkers levels [2].

Variable	Non-Current (Ref)	Current, CPD < 10				Current, CPD ≥ 10				
	N	*N*	Beta	SE	*P*	*N*	Beta	SE	*P*	P_{trend}
Haptoglobin	81	12	0.17	0.15	0.26	17	0.42	0.13	0.001	0.001
CCL17	81	12	0.24	0.18	0.20	17	0.47	0.16	0.003	0.002
Baff	87	12	−6.78	55.53	0.90	17	165.80	47.23	0.0004	0.002
sgp130	87	12	−10.57	7.65	0.17	17	−13.06	6.53	0.05	0.02
MCP1	87	12	0.22	0.16	0.19	17	0.07	0.14	0.60	0.42

[1] Adjusted for age and sex in the mixed models. Except BAFF and gp130, all the biomarkers were run as log2 transformed in the mixed models. [2] The unit of all biomarkers is pg/mL, except sgp130 as ng/mL.

Table S7. Biomarker intra-pair correlation coefficient (ICC) in the 67 MZ twin pairs.

Biomarker	ICC (95%CI)	*P*
IL6RA	0.90(0.84–0.94)	7.05×10^{-26}
CCL24	0.87(0.80–0.92)	4.96×10^{-23}
EndoCAB	0.70(0.56–0.81)	3.11×10^{-11}
ICAM	0.67(0.51–0.78)	2.18×10^{-10}
TNFRII	0.66(0.50–0.77)	4.77×10^{-10}
CCL17	0.66(0.50–0.78)	6.69×10^{-10}
LBP	0.65(0.48–0.77)	1.07×10^{-9}
IL2Ra	0.62(0.45–0.75)	6.25×10^{-9}
BAFF	0.60(0.42–0.73)	3.00×10^{-8}
IL1Ra	0.55(0.37–0.70)	3.95×10^{-7}
CCL22	0.55(0.37–0.70)	4.02×10^{-7}
CD163	0.54(0.35–0.69)	6.81×10^{-7}
sgp130	0.54(0.35–0.69)	8.81×10^{-7}
CD30	0.53(0.34–0.68)	1.42×10^{-6}
IL18	0.52(0.32–0.68)	2.37×10^{-6}
CD14	0.51(0.31–0.67)	4.47×10^{-6}
FGF21	0.49(0.28–0.65)	1.22×10^{-5}
IL8	0.45(0.24–0.62)	4.64×10^{-5}
IP10	0.44(0.23–0.62)	6.77×10^{-5}
Haptoglobin	0.44(0.22–0.62)	0.0001
IL15	0.41(0.19–0.59)	0.0002
FABP2	0.41(0.19–0.59)	0.0002
IL6	0.42(0.18–0.61)	0.001
FABP4	0.34(0.12–0.54)	0.002
CXCL13	0.34(0.11–0.53)	0.002
MCP1	0.28(0.04–0.48)	0.01
TNFa	0.14(−0.11–0.38)	0.13

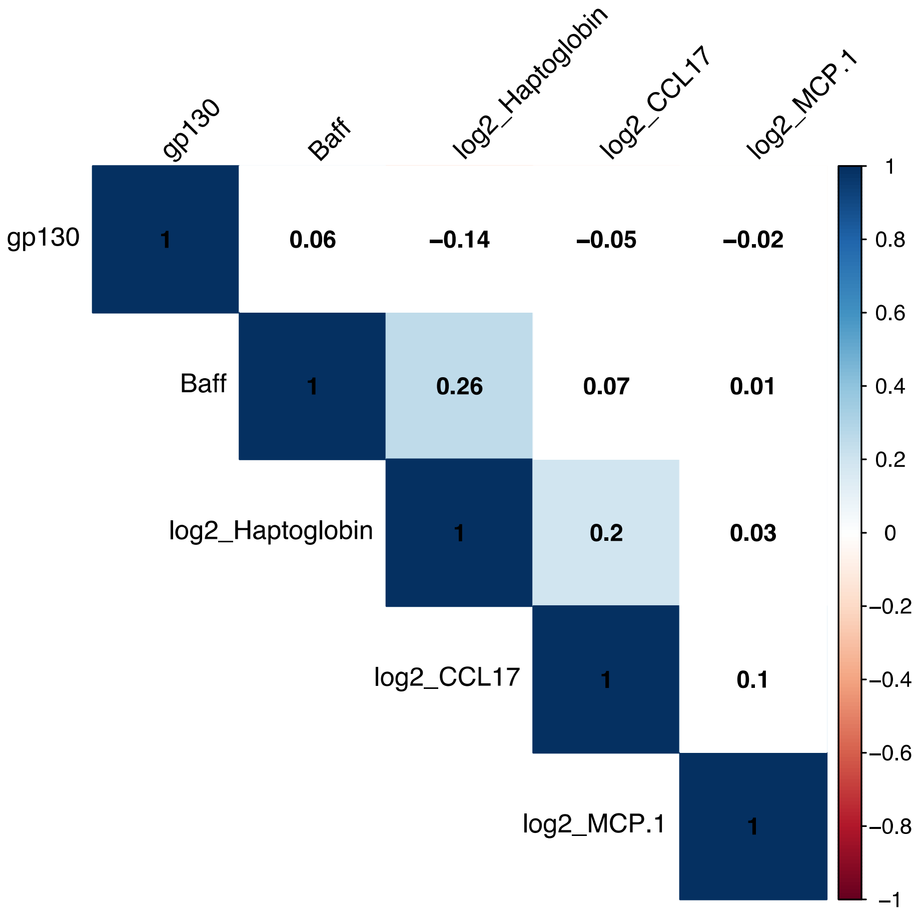

Figure S1. Pearson correlation [1] of serum biomarkers [2]. [1] Numbers are correlation coefficients. Non-significant correlation ($P > 0.05$) is shown as white color. [2] Only for markers which were significant association with serum cotinine levels.
